# A Purine Analog Synergizes with Chloroquine (CQ) by Targeting *Plasmodium falciparum* Hsp90 (PfHsp90)

**DOI:** 10.1371/journal.pone.0075446

**Published:** 2013-09-30

**Authors:** Dea Shahinas, Asongna Folefoc, Tony Taldone, Gabriela Chiosis, Ian Crandall, Dylan R. Pillai

**Affiliations:** 1 Department of Laboratory Medicine & Pathobiology, University of Toronto, Toronto, Ontario, Canada; 2 Memorial Sloan-Kettering Cancer Center New York, New York, United States of America; 3 Department of Pathology & Laboratory Medicine and Medicine, The University of Calgary, Calgary, Alberta, Canada; 4 Department of Microbiology, Immunology, and Infectious Diseases, The University of Calgary, Calgary, Alberta, Canada; Johns Hopkins Bloomberg School of Public Health, United States of America

## Abstract

**Background:**

Drug resistance, absence of an effective vaccine, and inadequate public health measures are major impediments to controlling *Plasmodium falciparum* malaria worldwide. The development of antimalarials to which resistance is less likely is paramount. To this end, we have exploited the chaperone function of *P. falciparum* Hsp90 (PfHsp90) that serves to facilitate the expression of resistance determinants.

**Methods:**

The affinity and activity of a purine analogue Hsp90 inhibitor (PU-H71) on PfHsp90 was determined using surface plasmon resonance (SPR) studies and an ATPase activity assay, respectively. In vitro, antimalarial activity was quantified using flow cytometry. Interactors of PfHsp90 were determined by LC-MS/MS. *In vivo* studies were conducted using the *Plasmodium berghei* infection mouse model.

**Results:**

PU-H71 exhibited antimalarial activity in the nanomolar range, displayed synergistic activity with chloroquine *in vitro*. Affinity studies reveal that the PfHsp90 interacts either directly or indirectly with the *P. falciparum* chloroquine resistance transporter (PfCRT) responsible for chloroquine resistance. PU-H71 synergized with chloroquine in the *P.berghei* mouse model of malaria to reduce parasitemia and improve survival.

**Conclusions:**

We propose that the interaction of PfHsp90 with PfCRT may account for the observed antimalarial synergy and that PU-H71 is an effective adjunct for combination therapy.

## Introduction

Heat shock protein 90 (Hsp90) is important for normal growth and development in eukaryotes. In higher eukaryotes, cytosolic Hsp90 exists in the form of a multi-chaperone complex and exerts different roles in protein trafficking [1]–[4]. It is postulated that Hsp90 serves as a buffer by preventing cellular toxicity caused by misfolded and aggregated proteins in response to stress. Hsp90 is not involved in primary protein folding events but rather in client protein maturation [5], [6]. It provides a compensatory regulatory mechanism that maintains the functional conformation of regulatory proteins, including many involved in drug resistance [2]–[4], [7]. A number of independent studies have demonstrated that Hsp90 is essential in eukaryotes and that inhibition of Hsp90 activity by small molecules is lethal to transformed cells**.** Infection, transformation and neurodegeneration are all characterized by abnormal cell signaling, altered levels of expression and different protein interactions in the cell [8]–[15] and as such, Hsp90 has been identified as a target for infections [2]–[4], [16], [17], cancer [10], [11], [18]–[22] and Alzheimer’s disease [8], [9].

In particular, the ATPase activity of Hsp90 is essential for driving the chaperone cycle and directing binding, induction of the active conformation and release of its client proteins [1], [8]–[10], [12], [14], [15], [18], [19], [23]–[26]. A range of Hsp90 inhibitors that target the N-terminal ATP-binding domain by competition have been developed including natural product inhibitors such as geldanamycin (GA) and radicicol (Rad). GA, an ansamycin antibiotic, is the first selective Hsp90 inhibitor that has been shown to bind Hsp90 and interfere with heterocomplex formation [1], [14], [15]. Radicicol (Rad) is a macrocyclic lactone with anti-Hsp90 activity in cell culture, but it is not stable in serum and therefore has no in vivo activity [1], [14], [15]. Upon inhibition, Hsp90 client proteins cannot attain their active conformation and are degraded by the proteasome. Degradation of these proteins leads to growth arrest and apoptosis [1], [8], [9], [14], [15]. In addition, Hsp90 is an attractive drug target because it mediates a broad spectrum of essential interactions and signaling pathways. It provides a wide range of potentially beneficial effects and a decreased likelihood for developing resistance. Due to the toxicity caused by GA, Rad, and its derivatives in animal and human studies, alternative small molecule inhibitors have been sought. For example, 17-AAG and other Hsp90 inhibitors such as the purine analogue PU-H71 (MeSH: C526550) are currently under clinical evaluation for a number of cancer states [27].

We and other groups have previously shown that Hsp90 inhibitors synergize with conventional antimicrobials when used in combination [2]–[4], [28]. The *P. falciparum* cytosolic Hsp90 (PF07_0029, new PlasmoDB ID: PF3D7_0827900, PfHsp90) is known to be stress-inducible [7], [29] and expressed during the intra-erythrocytic life cycle of the parasite [30]. PfHsp90 is essential for the development of the parasite [23]–[25], [31]. Targeting PfHsp90 therefore affords the possibility of developing a synergistic adjunctive therapy by inhibiting the folding of key antimalarial resistance determinants. In a recent study, Pallavi *et al.* also implicated Hsp90 as a drug target against malaria and *Trypanosoma* infection in animal models [32]. Biochemical characterization of full length PfHsp90 in this study showed that PfHsp90 retains high level ATPase activity which was inhibited successfully using 17-AAG. 17-AAG reduced parasite infection load in the *P.berghei* model at the tested dose of 50 mg/kg. In order to obtain drugs with fewer side effects, the purine scaffold inhibitors have been exploited to take advantage of the unique shape that ATP conforms to when binding the ATP-binding domain [1], [14], [15]. The structure of the first ATP mimetics was used for extensive chemical modification in order to enhance potency [1], [14], [15], [19]. One of the most potent representatives of this group with an attractive pharmacokinetic profile is PU-H71 [1], [14], [15], [19]. We hypothesized that PU-H71 may serve well as an antimalarial by targeting Hsp90. We show here that PU-H71 binds with high affinity to the PfHsp90 ATP-binding domain and inhibits ATPase activity. PU-H71 exhibits potent antimalarial activity both in cell culture and in the *Plasmodium berghei* model of malaria and was able to synergize with chloroquine (CQ). Based on biochemical and genetic studies we postulate that the effect may be in part related to a direct association between PfHsp90 and the *P. falciparum* Chloroquine Resistance Transporter (PfCRT). We conclude that PU-H71 synergized with CQ to reduce parasite load and improve rates of survival in the *P.berghei* Balb/c mouse model.

## Materials and Methods

### PU-H71 and Geldanamycin (GA) Docking in the PfHsp90 GHKL Domain Structure

The GA and PU-H71 docking models were generated using the PfHsp90 crystal structure template (PDB ID: 3K60) and the HsHsp90 co-crystal structure with PU-H71 (PDB ID: 2FWZ). The docking was done using Coot 0.1 [33]. Visualization of the model and preparation of the figures was performed with PyMOL 1.1 and Adobe Photoshop CS4.

### Surface Plasmon Resonance (SPR) Measurements

All SPR measurements were conducted on a Biacore × instrument (GE Healthcare, Waukesha, WI) at 25°C as described previously [34]. The recombinant PfHsp90 ATP binding domain was purified as previously described [28] and was immobilized to one of two flow cells on a CM5 chip (GE Healthcare, Waukesha, WI) using an amine coupling kit (GE Healthcare, Waukesha, WI) as per manufacturer’s protocol. The second flow cell was sham activated and deactivated and was used as a reference for the refractive index changes during the experiment.

Immobilizations and binding observations were performed using a flow buffer that consisted of: 10 mM HEPES pH 7.5, 150 mM KCl, 3 mM EDTA, 0.005% P20 surfactant. 12000 response units (RU) of immobilized protein were obtained with a 30 µL injection of 100 µg/mL protein in 10 mM sodium acetate pH 4.0. For binding experiments with a range of PU-H71 concentrations (5 µM–1.25 mM), the 5 mM drug stock was diluted in the flow buffer and was run over the surface of the chip at a flow rate of 5 µL/min with 40 µL injections. When necessary, 2.0 M NaCl pH 5.0 was used to dissociate PU-H71 and restore the baseline for the subsequent concentration of drug. The steady state responses were plotted against the PU-H71 concentrations. To obtain the dissociation constant, these responses were fit to a 1∶1 Langmuir binding model by nonlinear regression using the BiaEvaluation 4.1 software (GE Healthcare GE Healthcare, Waukesha, WI).

### ATPase Activity Assay

Recombinant full length Hsp90 was expressed and purified as previously described [32]. ATPase activity of full length Hsp90 was measured using the coupled LDH/NADH method as previously described [35]. Briefly, the reaction mixture was set up in a final volume of 100 µL containing 25 mM HEPES, 5 mM MgCl_2_, 5 mM KCl, 3 µM Hsp90, 5 mM ATP, 0.2 mM NADH, 3 mM phosphoenol pyruvate, 4.7 U pyruvate kinase, 7.4 U lactate dehydrogenase, 0.03% Tween 20, and 10% glycerol. The decrease in NADH absorbance at 340 nm was recorded continuously for 40 minutes using an EnSpire multimode reader (Perkin Elmer, Waltham, MA). The mixture without the ATPase was incubated at 37°C for 2 minutes and then NADH absorbance was recorded for 3 minutes and used as background to account for spontaneous ATP hydrolysis. The NADH absorbance was recorded for 20 minutes continuously at an acquisition rate of 3 readings per minute at the wavelength of 340 nm.

### *P. falciparum* Culture Methods and Antimalarial Cell Assay

The *P. falciparum* strains 3D7, W2 and Dd2 were grown in 5% hematocrit and RPMI 1640 medium supplemented with 0.25% Albumax II, 2 g/L sodium bicarbonate, 0.1 mM hypoxanthine, 25 mM HEPES (pH 7.5), 50 g/L gentamycin at 37°C, 5% O_2_, and 6% CO_2_.Growth inhibition of *P. falciparum* cultures was quantified using a flow cytometric assay. The cultures were synchronized with 5% sorbitol and grown for 48 hours. They were diluted to 1.0% rings and 0.5% hematocrit and cultured in the presence of each of the drugs or their combinations for 72 hours. The cultures were stained for 1 h at room temperature (RT) with 1×SYBR Green in phosphate buffered saline (PBS) pH 7.4 solution. Samples were analyzed with a Cytomics FC-500 MPL flow cytometer, (Beckman Coulter, Miami, FL). To evaluate the antimalarial activity of combinations of lumefantrine, quinine and amodiaquine with PU-H71, the histidine rich protein II (HRPII) ELISA protocol was followed as per Noedl *et al*. [36]. Inhibition of parasite growth in each sample was evaluated relative to infected erythrocytes without drug treatment (PBS alone). To establish the rings and mature flow cytometry gates, the parasites were sampled at the time points of 0, 6, 12, 24, 36 and 48 hrs. To test the heat shock response, the experimental schema was adapted from Pavithra et al. [31]. The parasites were heat shocked for 2 hours at 40°C. The temperature was restored to 37°C for 10 hours and parasites were then heat shocked for another 10 hours at 40°C. Rings and trophozoites were distinguished based on the gating established for the different points of the *P. falciparum* intra-erythrocytic cycle described above.

### *In vitro* PU-H71 and Chloroquine Drug Interactions

Concentrations for the drug interaction experiments were selected to be lower than the IC_50_s of each drug tested alone. IC_50_s and fractional IC_50_s (FIC_50_) were derived by dose-response curve fitting of the data from duplicate 72-hr assays. FIC_50_ was determined using the formula IC_50_ (A+ fixed [B])/IC_50_ of A alone. Average sum FIC_50_ was determined by the sum of the ratio of the IC_50_ of each drug in combination treatments over the IC_50_ of each of the drug administered alone (ie. IC_50_ (A+ fixed [B])/IC_50_ of A alone+IC_50_ (B+ fixed [A])/IC_50_ of B alone). Synergistic activity was defined by a sum FIC ratio ≤0.5. In addition, the FIC_50_ values were used to plot isobolograms, which display synergistic activity of the drugs if the points lie below the diagonal line that joins the FIC_50_ points of 1 on each of the axes [37]. To determine the CQ effect with PU-H71 in both the CQ sensitive parasite line 3D7 and the CQ resistant line W2, the response modification index (RMI) was calculated using the formula IC_50_ (CQ+PU-H71)/IC_50_(CQ) [38], [39]. An RMI of ≈1 denotes no change in antimalarial activity, whereas an RMI <<1 represents potentiation of antimalarial activity (ie. synergistic activity) and an RMI>>1 is a sign of antagonistic activity [38], [39].

### Parasite Protein Extraction

*Plasmodium falciparum* W2 culture (800 mL, mixed stages of parasites) at an average parasitemia of 9% was used for protein extraction. To purify the intact parasites, the red blood cells were lysed with 0.1% saponin and washed with PBS until the solution was transluscent to eliminate most of the hemoglobin. The parasites were lysed on a nutator at 4°C for 1 h in 2 mL of: 10 mM Hepes pH 7.5, 1.5 mM MgCl_2_, 10 mM KCl, 10 µL benzonase, 10 µL protease inhibitor cocktail, 10 µL PMSF, 1% dodecylmaltoside (w/v). The lysed parasites were spun down at 14000 rpm for 30 minutes and the supernatant containing the soluble protein extract was used for co-immunoprecipitation.

### Co-immunoprecipitation of PfHsp90 and PfCRT

The rabbit anti-PfHsp90 antibody was obtained from StressMarq Biosciences (Victoria, BC) and the rabbit anti-PfCRT antibody was obtained from the malaria resource centre (MR4). The corresponding (PfHsp90 or PfCRT) antibody (16 µg) was conjugated to 200 µL of protein A/G/L sepharose beads slurry (BioVision, Mountain View, CA) by incubation for 2 h at room temperature on a rotator. After washing the unbound antibody with 0.1 M sodium borate pH 9.0, the antibody was conjugated to the beads with 20 mM dimethyl pimelimidate (DMP) in borate solution twice for 30 minutes. The beads were washed with 50 mM glycine at pH 2.5 and were further washed with PBS pH 7.4.

The conjugated antibody bead slurry as well as unbound beads (control) was incubated with 100 µg of the parasite protein extract for 4 hours under rotation at 4°C. After centrifugation, the unbound protein solution was removed and the beads were washed 2× with 1% SDS, 5 mM EDTA pH 8.0 solution (denaturing conditions). The beads were boiled in this solution at 95°C for 5 minutes and were further analyzed with SDS-PAGE (12%) and Western blotting using a dilution of 1∶2000 for the PfHsp90 antibody and 1∶1000 for the PfCRT antibody. For the non-denaturing condition elution, proteins bound to the beads were eluted with 0.1 Mglycine, pH 2.5, and the samples were neutralized by adding 1 M Tris-HCl pH 9.0, and analyzed by native PAGE.

For the recombinant PfHsp90-interactor pulldown experiment, 50 µg of histidine-tagged full length PfHsp90 was incubated overnight at 4°C with 500 µL of Ni-NTA Superflow (Qiagen, Germantown, MD) bead slurry equilibrated in binding buffer (50 mM Tris-HCl pH 7.5, 150 mM NaCl, 10% glycerol, 10 mM imidazole). The next day, the PfHsp90 bound bead slurry was incubated with 100 µg of the parasite protein extract for 2 h under rotation at 4°C. The beads were washed twice with 1% SDS, 5 mM EDTA pH 8.0. The beads were boiled in this solution at 95°C for 5 minutes and the supernatant was further analyzed with SDS-PAGE (12%) and Western blotting using a dilution of 1∶1000 for the PfCRT antibody.

### LC-MS/MS Analysis

Bands from silver-stained and Coomassie stained gels of the denatured reciprocal immunoprecipitation supernatants were cut and submitted for in-gel trypsin digestion and LC-MS/MS analysis at the Advanced Protein Technology Centre at the Hospital for Sick Children (Dr. Li Zhang,Toronto, ON). The following report was generated from Scaffold v3.2 to report the protocol followed during the analysis: MS/MS samples were analyzed using Mascot (Matrix Science, London, UK; version Mascot). Mascot was set up to search the NCBI nr_20110813 database (selected for *Plasmodium falciparum*, 18091 entries) with the digestion enzyme trypsin. Mascot was searched with a fragment ion mass tolerance of 0.40 Da and a parent ion tolerance of 20 PPM. Iodoacetamide derivative of cysteine was specified in Mascot as a fixed modification. Pyro-glu from E of the N-terminus, s-carbamoyl methyl cysteine cyclization (N-terminus) of the N-terminus, deamidation of asparagine and glutamine, oxidation of methionine and acetylation of the N-terminus were specified in Mascot as variable modifications. Scaffold (version Scaffold_3.2.0, Proteome Software Inc., Portland, OR) was used to validate MS/MS based peptide and protein identifications. Peptide identifications were accepted if they could be established at greater than 50.0% probability as specified by the Peptide Prophet algorithm [40]. Protein identifications were accepted if they could be established at greater than 80.0%probability and contained at least 1 identified peptide. Proteins that contained similar peptides and could not be differentiated based on MS/MS analysis alone were grouped to satisfy the principles of parsimony. The proteins identified were cross-referenced to PlasmoDB [41], [42] and the protein-protein interaction network was generated using Cytoscape v2.8 [43].

### *Plasmodium berghei* Mouse Model Studies and Drug Administration

The work was carried out under an Animal Use Protocol that was approved by the Faculties of Medicine and Pharmacy Animal Care Committee (FMPACC) at the University of Toronto. Animals were sacrificed by CO_2_ inhalation and during the experiment they were monitored for: failure to groom (piloerection), lethargy or reluctance to move, weight loss exceeding 20% of normal body weight, sunken eyes and pinched face indicative of advanced dehydration. Animals were monitored twice a day and the advice of a veterinarian was sought as required. BALB/c mice were ordered from the Jackson Laboratories (Bar Harbor, ME). Mice were infected intra-peritoneally (i.p.) with 10^6^ *Plasmodium berghei* ANKA strain parasites passaged in the laboratory of Dr. Kevin Kain. This mouse model of malaria was chosen because upon infection with this especially virulent ANKA strain, the mice can be monitored for signs of severe disease such as lethargy, ruffled fur and sunken eyes. In addition, the trajectory of the parasitemia changes in these models is predictable with the mice reaching a parasitemia of 1% 5 days after infection and cerebral malaria at day 13.

Any changes in response to drug treatment may be monitored accordingly. Following infection, mouse weight and parasitemia were monitored daily. The PU-H71 and CQ solutions were prepared fresh daily in PBS. Injections were performed intra-peritoneally (i.p.) for 3 days in a row after the parasitemia level reached 1%. Untreated control animals were treated with PBS alone. Synergistic drug interactions were determined using previously published definitions of antimalarial drug synergy in vivo, which postulate that two drugs are synergistic if the treatment with a combination of less or equal to half the ED_50_ for each drug results in parasitemia inhibition of ≈50% or more [37], [39]. Another published definition of drug synergism in vivo suggests that the percent inhibition of parasitemia by the combination treatment is 10% higher than the sum of the inhibition observed by each of the treatments alone at the same doses (i.e. % inhibition (A+B)>% inhibition (A)+% inhibition (B) +10% [44]).

## Results

### Homology Modeling Reveals that PU-H71 Fits Well in the PfHsp90 ATP Binding Domain

**Figure 1:**
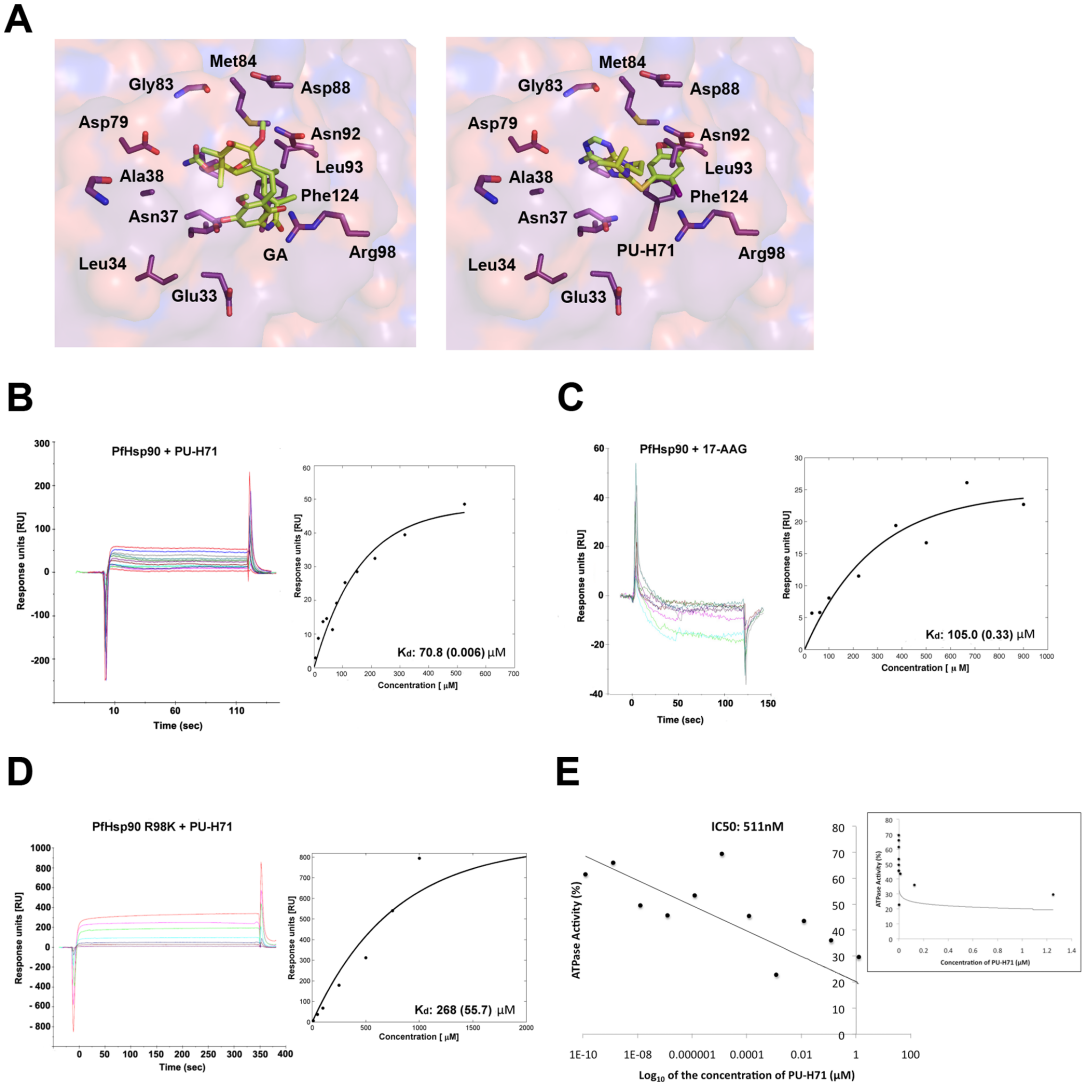
Biochemical evaluation of the affinity of PU-H71 for PfHsp90. (A) Illustration of geldanamycin (GA, left) and PU-H71 (right) docked within the ATP-binding site of PfHsp90. The models were generated using the PfHsp90 crystal structure (PDB ID: 3K60) [45]. By convention, the electrostatic potential surface in the background denotes acidic residues in red and basic residues in blue. (B) Surface plasmon resonance (SPR) measurements for PU-H71 binding to the ATP-binding domain of PfHsp90. The colors on the sensorgrams represent varying concentrations of the respective drug (5–1000 µM) injected over the surface with the immobilized PfHsp90. The steady state responses were fitted using non-linear regression to a single binding site model (as shown on the right) to obtain the K_d_ value indicated. The number in parentheses represents standard error on the K_d_ obtained from fits. The sensorgrams shown have been double referenced as described previously [34]. (C) 17-AAG was used as a positive control drug. (D) SPR measurements for PU-H71 binding of the R98K mutant ATP-binding domain of PfHsp90. (E) Full-length PfHsp90 was expressed and ATPase activity tested in the presence of PU-H71. Results are shown as a percentage of total ATPase activity in the absence of drug and IC_50_ indicated (511 nM) for a single experiment. Positive control drug treatments included radicicol (144 nM) and 17-AAG (146 nM). The inset shows the logarithmic curve fitting of ATPase activity with increasing concentration of PU-H71.

To determine whether PU-H71 fits into the PfHsp90 ATP-binding pocket, PU-H71 was docked in the crystal structure of the PfHsp90 ATP-binding domain (PDB ID: 3K60) (Figure 1A) [45]. The analysis supported a model where PU-H71 employs ionic interactions with the guanidinium group of arginine 98 (Arg98). The generic Hsp90 inhibitor, geldanamycin (GA), was also modeled in the malaria Hsp90 binding pocket. GA occupies a larger space of the pocket due to the ansamycin moiety.

### PU-H71 Binds the PfHsp90 ATP-binding Domain with High Affinity and Inhibits its ATPase Activity

To quantify the affinity of PU-H71 for the PfHsp90 ATP-binding domain, surface plasmon resonance experiments were employed using a Biacore × system. Titration of PU-H71 on the surface-immobilized PfHsp90 ATP-binding domain reached saturation (Figure 1B). A titration dependent effect was observed for PU-H71 binding of the PfHsp90 ATP-binding domain with a dissociation constant (K_d_) of 70.8±0.006 µM as compared to the positive control GA derivative 17-AAG with a Kd of 105±0.33 µM (Figure 1C). To test the importance that the Arg98 guanidinium group plays in accommodating PU-H71 in the PfHsp90 ATP binding pocket, a R98K site directed mutant of PfHsp90 ATP binding domain was immobilized on the surface of a Biacore × chip. PU-H71 bound this immobilized R98K mutant PfHsp90 surface with a K_d_ of 268±55.7 µM (Figure 1D). Full-length PfHsp90 was also expressed to test the ability of PU-H71 to inhibit the PfHsp90 ATPase activity. PU-H71 inhibited ATPase activity with an IC_50_ of 511 nM (Figure 1E). Drug positive controls included radicicol (144 nM) and 17-AAG (146 nM).

### PU-H71 Exhibits Anti-malarial Activity in *P. falciparum* Cell Culture and Acts Synergistically with Chloroquine and Lumefantrine

**Figure 2:**
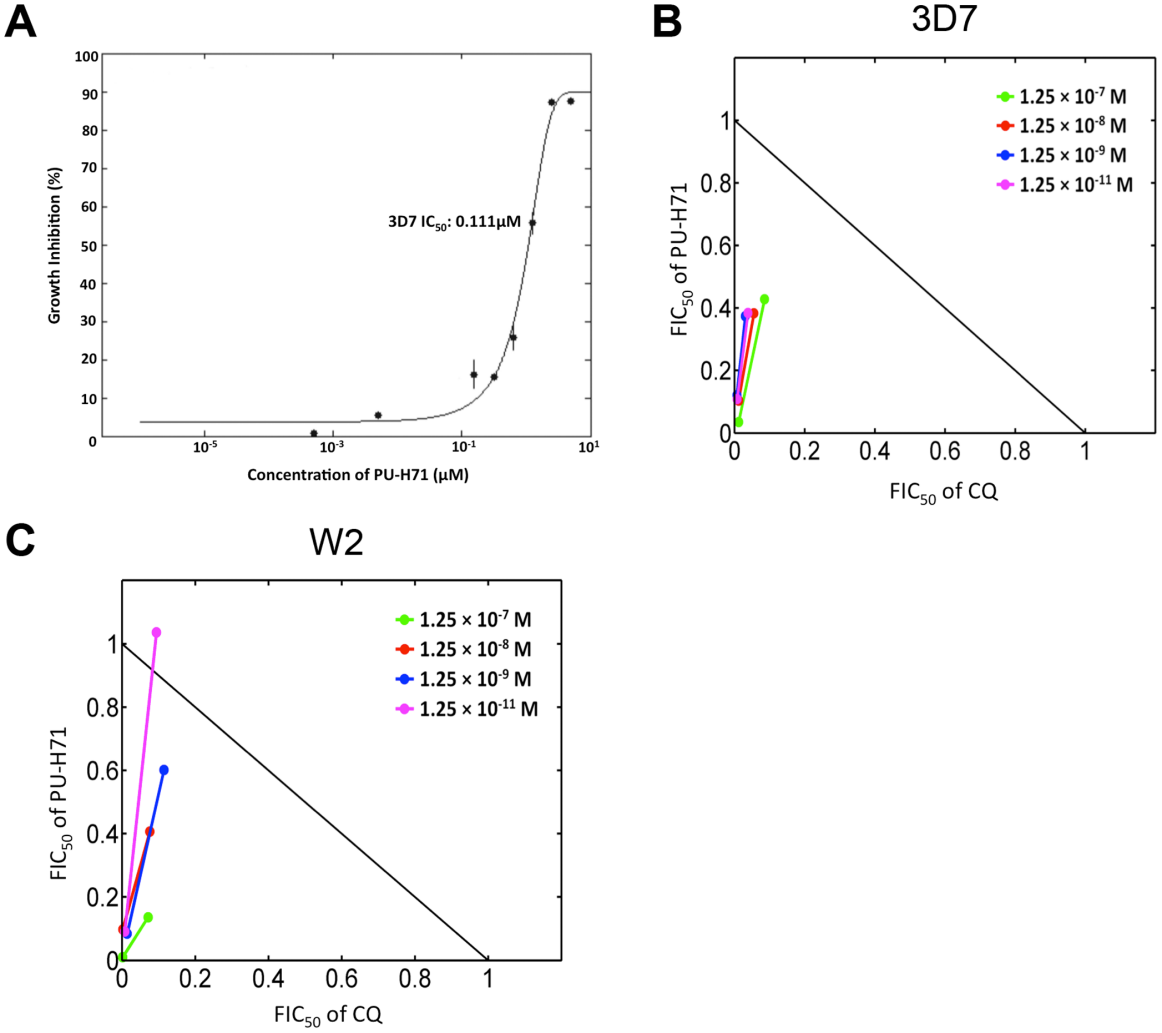
Assessment of the *in vitro* antimalarial activity of PU-H71. (A) Antimalarial activity of PU-H71 using a standard SYBR Green cell-based assay using the laboratory strain 3D7 resulted in an IC_50_ of 89.4±15.3 (SEM) nM (n = 3, performed in duplicate). A single representative experiment performed in duplicate is depicted here resulting in a IC_50_ of 111±8 nM (SD). Antimalarial activity of PU-H71 using the strain W2 resulted in an IC_50_ of 94.3±6.3 (SEM) nM. (B) PU-H71 acts synergistically with chloroquine (CQ) in a CQ sensitive strain (3D7). Synergistic activity was defined by calculations of the average sum fractional inhibitory concentration ratio (FIC) FIC_50_ and by the FIC_50_ ratios lying under the line corresponding to additive activity. (C) PU-H71 acts synergistically with chloroquine (CQ) and potentiates CQ activity in the CQ resistant strain W2. The dots on the isobolograms represent the average of technical duplicates. The calculations for the FIC ratios are enclosed in Table S2. The response modification index (RMI) was calculated for each of the doses tested in both 3D7 and W2 strains and has been summarized in Table 1.

To determine the antimalarial potency of PU-H71, a cell-based anti-malarial assay was performed based on flow cytometric measurements of SYBR Green staining *P. falciparum*-infected red blood cells. PU-H71 demonstrated a 50% inhibitory concentration (IC_50_) of 111 nM±8 nM (Figure 2A) in the CQ-sensitive strain 3D7. PU-H71 was evaluated for synergy in combination with the antimalarial chloroquine (CQ), lumefantrine, quinine, and amodiaquine. Isobolograms for CQ and PUH71 were generated for the synergistic combination of each drug in the parasite strain 3D7 (Figure 2B) and in the CQ-resistant strain W2 (Figure 2C). Fractional inhibitory concentration (FIC) ratios were calculated as previously described, with synergy defined by an average sum FIC ratio for both drugs of ≤0.5 and summarized for all other antimalarial drugs tested in Table 1 and Table 2 [46]. Quinine is synergistic in Dd2 and additive in 3D7. Amodiaquine is synergistic or additive (depending on concentration) in Dd2 but antagonistic in 3D7. Lumefantrine is synergistic in both 3D7 and Dd2. We also analyzed whether PU-H71 was able to reverse resistance in the CQ resistant strain W2 as defined by the ability to restore CQ susceptibility to the IC_50_ range typically seen with the susceptible strain *P. falciparum* strain (3D7) and by a response modifciation index (RMI) <<1 to represent potentiation of CQ antimalarial activity (Table 3). In checkerboard assays, a range between 0.4–0.5 sum FIC ratios was observed for 3D7 in the concentration range 1.25×10^−11^M to 1.25×10^−7^ M for each of the drugs PU-H71 and CQ (Figure 2B). With the CQ-resistant line W2, a sum FIC ratio range between 0.2–1.0 in the concentration range 1.25×10^−11^ M to 1.25×10^−7^ M. The RMI for CQ using the above drug combinations was in the range of 0.02–0.04 for 3D7 and 0.04–0.06 for W2 (Table 1). This index represents the extent to which PU-H71 modifies the CQ IC_50_.

**Table 1:** Synergy studies conducted with current antimalarials used in malaria endemic regions in the CQ sensitive strain 3D7.

Combination drug	Combination drug concentration (nM)	PU-H71 concentration (nM)	FIC
Quinine	1.25	0.000000125–1.25	1.49
	0.125	0.000000125–1.25	1.49
	0.0125	0.000000125–1.25	1.49
	0.00125	0.000000125–1.25	1.37
	0.000125	0.000000125–1.25	1.32
	0.0000125	0.000000125–1.25	0.30
	0.00000125	0.000000125–1.25	0.29
	0.000000125–1.25	0.125	0.0005
	0.000000125–1.25	0.000125	0.002
	0.000000125–1.25	0.000000125	0.003
Amodiaquine	0.0000125	0.000000125–1.25	5.29
	0.00000125	0.000000125–1.25	5.83
	0.000000125–1.25	0.0000125	0.002
Lumefantrine	0.125	0.000000125–1.25	0.003
	0.0125	0.000000125–1.25	0.003
	0.000000125–1.25	0.0000125	0.003
	0.000000125–1.25	0.00000125	0.003

* Shaded areas indicate the concentration of the drug that was kept constant, while the combination drug concentration was varied to determine the corresponding IC50.

**Table 2:** Synergy studies conducted with current antimalarials used in malaria endemic regions in the CQ resistant strain Dd2.

Combination drug	Combination drug concentration (nM)	PU-H71 concentration (nM)	FIC
Quinine	1.25	0.000000125–1.25	1.07
	0.125	0.000000125–1.25	0.34
	0.0125	0.000000125–1.25	0.35
	0.00125	0.000000125–1.25	0.36
	0.000125	0.000000125–1.25	0.37
	0.0000125	0.000000125–1.25	0.37
	0.00000125	0.000000125–1.25	0.35
	0.000000125	0.000000125–1.25	0.33
	0.000000125–1.25	1.25	0.002
	0.000000125–1.25	0.125	0.014
	0.000000125–1.25	0.0125	0.001
	0.000000125–1.25	0.00125	0.001
Amodiaquine	0.0125	0.000000125–1.25	0.66
	0.00125	0.000000125–1.25	0.48
	0.000125	0.000000125–1.25	0.54
	0.0000125	0.000000125–1.25	0.61
	0.00000125	0.000000125–1.25	0.47
	0.000000125	0.000000125–1.25	0.57
	0.000000125–1.25	0.0125	0.002
	0.000000125–1.25	0.000125	0.004
	0.000000125–1.25	0.0000125	0.004
	0.000000125–1.25	0.00000125	0.035
Lumefantrine	0.125	0.000000125–1.25	0.003
	0.0125	0.000000125–1.25	0.005
	0.00125	0.000000125–1.25	0.004
	0.000125	0.000000125–1.25	0.004
	0.0000125	0.000000125–1.25	0.004
	0.00000125	0.000000125–1.25	0.003
	0.000000125–1.25	0.0125	0.142
	0.000000125–1.25	0.00125	0.003

* Shaded areas indicate the concentration of the drug that was kept constant, while the combination drug concentration was varied to determine the corresponding IC50.

**Table 3:** Summary of response modification indexes (RMI) for various combinations of PU-H71 and chloroquine *in vitro* based on the checkerboard assay.

Genetic Background	3D7	W2
Concentration of each drug	Response modification index*	Response modification index*
1.25×10^−7^M	0.04±0.037	0.04±0.035
1.25×10^−8^M	0.03±0.022	0.04±0.037
1.25×10^−9^M	0.02±0.012	0.06±0.050
1.25×10^−11^M	0.02±0.015	0.05±0.043

* A response modification index (RMI) of ≈1 correspond with no effect of one drug on another when used in combination; RMI <<1 potentiation of antimalarial activity (ie. synergistic activity); RMI>>1 antagonistic activity [38], [39]. Standard error of the mean is indicated.

### Immunoprecipitation Studies Demonstrate an Association between PfHsp90 and PfCRT

**Figure 3:**
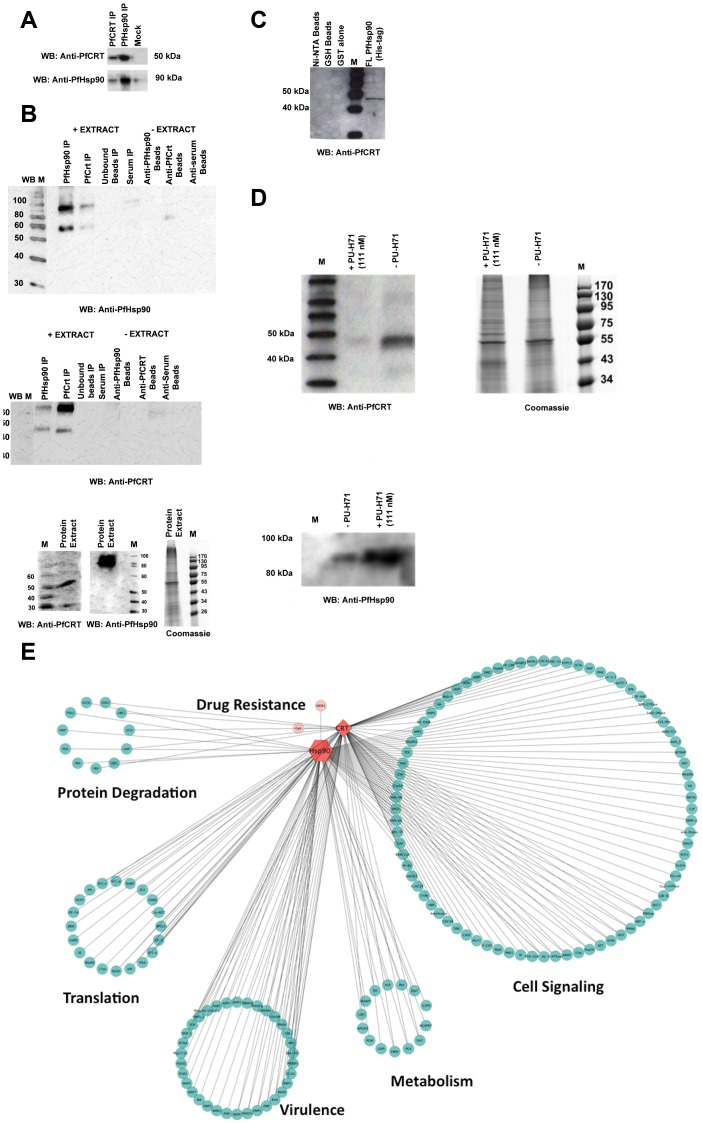
Interaction of PfHsp90 with PfCRT is demonstrated using a combination of co-immunoprecipitation, LC-MS/MS and, and PfHsp90 inhibition studies. The Coomassie stained gels are shown to indicate loading. (A) Under non-denaturing conditions, immunoprecipitation with anti-PfHsp90 (upper panel) pulled down both itself and the PfCRT as demonstrated by western blot. The converse experiment (with anti-PfCRT immunoprecipitation (lower panel) achieved the reciprocal result. The mock control used was beads alone plus extracts. (B) Anti-PfCRT (top) and anti-PfHsp90 (middle) Western blots of immunoprecipitates under denaturing conditions with associated controls. These western blots are displayed as composites because they were run on two separate gels. The exposure levels were matched. The lowermost panel displays western blots of both PfHsp90 and PfCRT from *P. falciparum* culture protein extracts. (C) Anti-PfCRT Western blot of proteins which bound to histidine-tagged full length (FL) PfHsp90 coupled to Ni-NTA beads. (D) Extracts from *P. falciparum* strain W2 treated and untreated for 24 hours with 111 nM PU-H71 and immunoblotted with anti-PfCRT and anti-PfHsp90 antibody to determine the level of PfCRT and PfHsp90 available following PfHsp90 inhibition. The upper right panel demonstrates equal loading of protein in drug treated and untreated fractions. (E) Unweighted protein-protein interaction network of LC-MS/MS analyzed immunoprecipitates using anti-PfHsp90 and anti-PfCRT. The complete list of constituents in the interactome is available in Table S1. The image was generated using Cytoscape v2.8 [43]. Lines connecting proteins suggest a direct interaction.

To determine whether the mechanism of the synergism with CQ might be related to an interaction between PfCRT and the PfHsp90 complex, we performed co-immunoprecipitation of W2 parasite protein extracts with antibodies specific to PfHsp90 (StressMarq, rabbit polyclonal IgM) or PfCRT (MR4, polyclonal rabbit serum). Immunodetection of PfCRT was observed following anti-PfHsp90 immunoprecipitation under both non-denaturing (Figure 3A) and denaturing conditions (Figure 3B). In the reciprocal experiment, PfHsp90 was immunodetected following immunoprecipitation when anti-PfCRT conjugated to sepharose beads was used with malaria extracts under both non-denaturing (Figure 3A) and denaturing conditions. Immunoprecipitation blots stained with anti-PfCRT antibody and anti-PfHsp90 antibody were stripped and hybridized with anti-human hemoglobin antibody (Cedarlane, mouse monoclonal IgG) and the secondary band at 60 kDa deemed to be hemoglobin. In order to further confirm the direct association between PfCRT and PfHsp90, histidine tagged full length PfHsp90 was bound to Ni-NTA Superflow beads (Qiagen, Germantown, MD) and the bound beads were incubated with W2 parasite extracts. PfCRT was immunodetected from the denatured pulled down proteins (Figure 3C). If PfHsp90 is responsible for folding PfCRT, then with PfHsp90 inhibition, the levels of PfCRT protein should decrease, because misfolded protein would likely be targeted for degradation. To test this, *P. falciparum* W2 parasites were treated with 111 nM PU-H71 (IC_50_ concentration) for 24 h. Inhibition of PfHsp90 by PU-H71 resulted in loss of PfCRT protein as would be expected based on Hsp90 client proteins being targeted for degradation upon its inhibition [47], [48] (Figure 3D).

### LC-MS/MS Analysis of Co-immunoprecipitated Proteins Confirms the Association of PfCRT and PfHsp90

Analysis by LC-MS/MS of co-immunoprecipitation gel bands subjected to tryptic digestion identified a total of 148 proteins from the anti-PfCRT pulldown and 187 proteins from the anti-Hsp90 pulldown (Table S1). Apart from confirming the association between PfHsp90 and PfCRT, several of the identified proteins were known interactors of PfHsp90 and PfCRT based on previous protein-protein interaction studies, key among which are the association of PfHsp90 with PfHsp70, PfHsp60, cyclophilin, Hsp70/Hsp90 organizing protein (HOP) etc [7], [49]. The protein interactions were visualized and clustered by molecular function using Cytoscape and Gene Ontology (GO) (Figure 3E). As expected, apart from a major cell signalling cluster (28.1% of all proteins identified) consisting of protein kinases and transcription factors, a virulence cluster (16.7%) including major factors such as erythrocyte membrane protein 1 (EMP1), apical membrane antigen 1 (AMA1), erythrocyte binding antigen 165 (EBA-165), merozoite surface protein 1 and 7 (MSP1, MSP7) etc. and other drug resistance associated proteins apart from PfCRT were part of the constructed network such as: multidrug resistance transporter 1 (MDR1) and Cg4.

### Resistance Selection Results in an ATP Binding Domain Mutant with Elevated PU-H71 IC_50_

**Figure 4:**
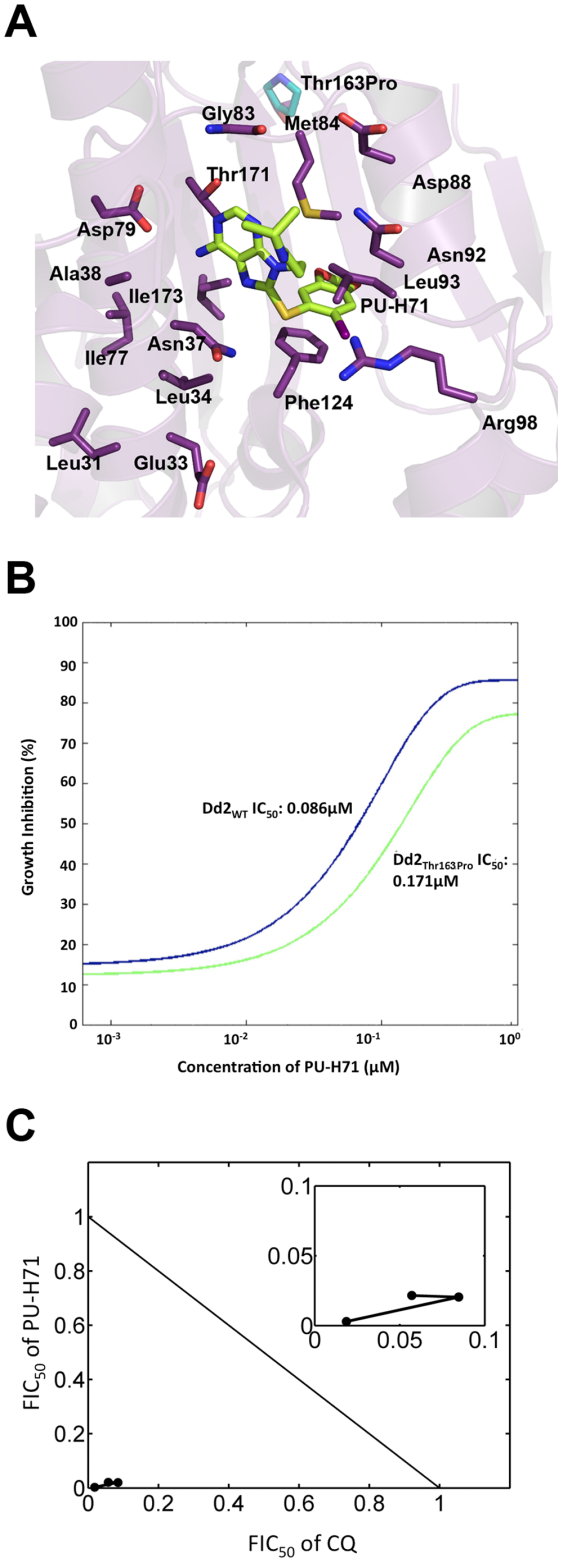
A step-wise selection method obtained the PfHsp90 Thr163Pro mutant (strain Dd2). (A) The Thr163Pro mutation is depicted computationally in the crystal structure of the PfHsp90 ATP-binding domain [45]. (B) The presence of this mutation resulted in doubling of the PU-H71 IC_50_ in the cell-based assay (a single representative experiment performed in duplicate is shown). (C) Synergy between PU-H71 and CQ in the Thr163Pro mutant was still observed based on sum FIC ratio ≤0.5. The inset provides a magnified view of the FIC_50_ ratios for the different PU-H71 and CQ drug combinations in the mutant strain.

If the PfHsp90 ATP binding domain is the specific target of Hsp90 inhibitors, then a mutation in this domain would be expected to arise in response to drug selection pressure. In order to test this hypothesis, we implemented a step-wise resistance selection method (based on Sidhu et al. [50]). Using this method, 1.25×10^8^ parasites underwent selection with increasing concentrations of the Hsp90 inhibitor (APPA) [28] ranging from 0.5× to 5× IC_50_ concentrations in a step-wise fashion. One resistant line was obtained upon treatment after 12 weeks. DNA sequencing analysis of theHsp90 ATP-binding domain showed that this mutant line contains a Thr163Pro mutation. This mutation was located close to the ATP-binding pocket as shown by homology modeling (Figure 4A). The IC_50_ of PU-H71 was elevated from 86 nM in the unselected Dd2 strain to 171 nM in the mutant Dd2^T163P^ strain (Figure 4B). This experiment was repeated twice. Despite the elevation in the IC_50_ of PU-H71, synergy, as defined by the calculated FIC and isobologram analysis, was still observed between CQ and PU-H71 (Figure 4C).

### PU-H71 Exhibits a Significant Antimalarial Effect in Mouse Models of Malaria Without Significant Toxicity

**Figure 5:**
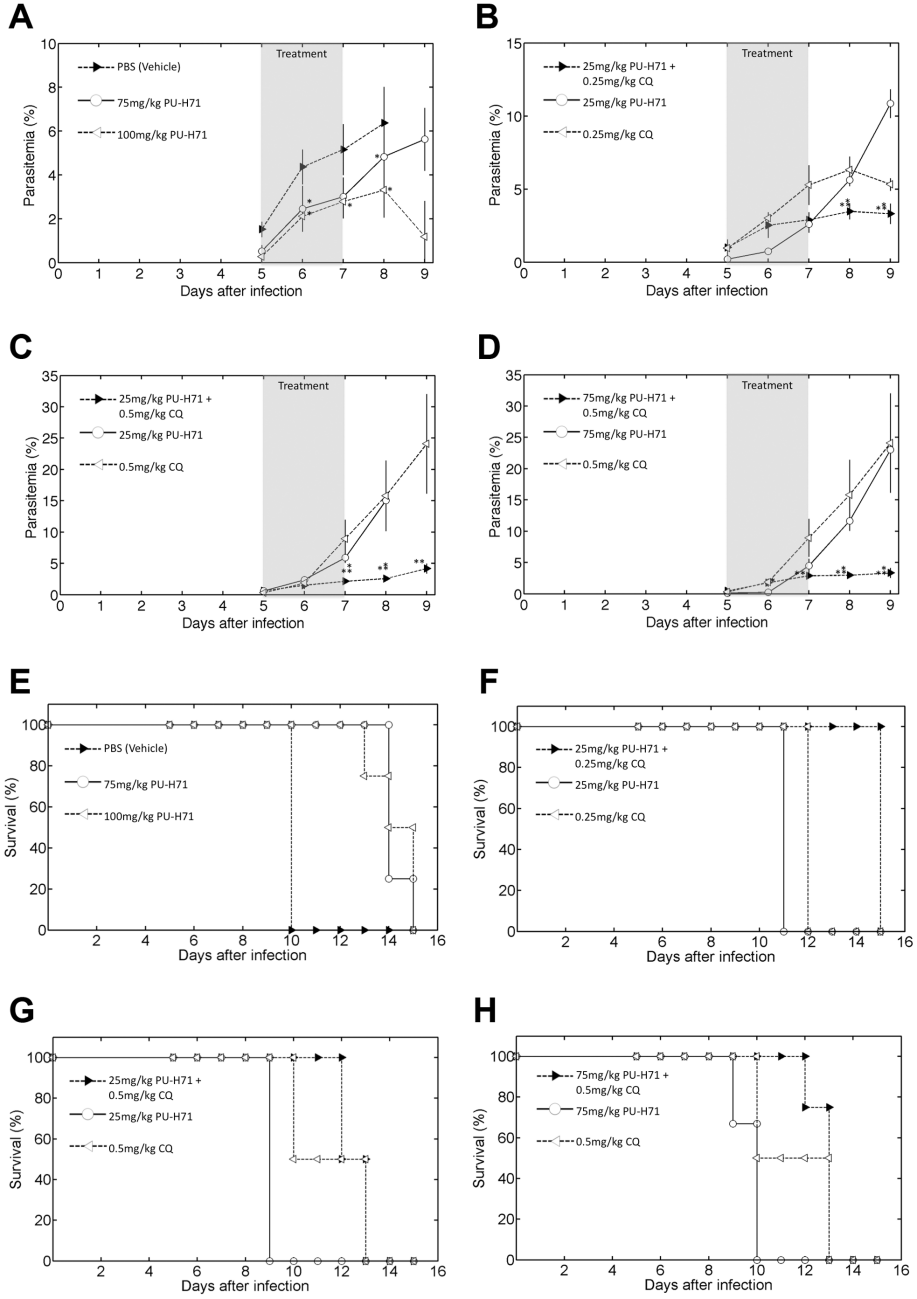
The effect of intra-peritoneal PU-H71 administration on the *Plasmodium berghei* mouse model. Mice were infected on day 0 and treatment was started on day 5 at a parasitemia of 1%. (A–D) Average parasitemia levels in response to drug treatments alone or in combination with CQ are indicated (n = 4 animals). The asterix (*) identifies significant differences in parasitemia relative to the vehicle control (A) and PU-H71 treatment alone (B–D) (Student’s t-test p≤0.05). The double asterix (**) indicates significant differences in parasitemia relative to the CQ treatment alone (Student’s t-test p≤0.05). (E-H) Kaplan-Meier survival plots for PU-H71 alone or in combination with CQ versus placebo.

To evaluate the antimalarial effect of PU-H71 in animals, *Plasmodium berghei* ANKA (10^6^ parasites) infected BALB/c mice were used. Daily intraperitoneal injections with PU-H71 were commenced once the parasitemia (i.e. the percent infected red blood cells) reached ∼1% (Day 5). Parasitemia was monitored by microscopic examination of Giemsa-stained thin blood smears prepared daily throughout the experiment. CQ (30 mg/kg) was used as the positive control and PBS was used as a vehicle control. Average parasitemia levels in response to drug treatments alone or in combination with CQ are shown in Figure 5 (A–D). Kaplan-Meier survival plots for PU-H71 alone or in combination with CQ versus placebo are shown in Figure 5 (E–H). Treatment of mice with 75 mg/kg and 100 mg/kg PU-H71 showed a significant protective effect in terms of reduced parasite burden (p≤0.05, unpaired t-test) and improved survival relative to the PBS control group (log-rank test), respectively (Figure 5A, 5E). The highest reduction in parasitemia was observed with three daily intraperitoneal injections of 100 mg/kg PU-H71. At day 8 (one day after the complete treatment was over), the group treated with 100 mg/kg was marked by an average of 46.3±10.8% inhibition of parasitemia relative to the vehicle. The combination of 25 mg/kg PU-H71 with 0.25 mg/kg CQ resulted in a significant (*p*<0.05, parametric unpaired t-test) average parasitemia reduction of 42.6±9.4% and improved survival by 3 days (Figure 5B, 5F) compared to 0.25 mg/kg CQ treatment alone. Using published definitions of synergism in vivo (described in Materials and Methods), the combination of 25 mg/kg PU-H71 (∼4×less than ED_50_) and 0.25 mg/kg CQ (∼5×lower than ED_50_) are the lowest doses at which synergistic activity was achieved. Other combinations were also able to achieve synergy (Table 4). Treatment with 2.5, 25, 75 and 100 mg/kg PU-H71 alone resulted in 15.8%, 11.7%, 24.2% and 46.3% inhibition of parasitemia on day 8, respectively. Combination treatments 0.25 mg/kg CQ +2.5 mg/kg PU-H71 and 0.25 mg/kg CQ +25 mg/kg PU-H71 resulted in 30.6% and 42.6% reduction in parasitemia on day 8, respectively. Complete elimination of parasites was not observed with any of those treatments. There was no significant toxic effect observed with any of the treatment regimens using PU-H71 both in infected mice and uninfected controls as evaluated by weight, cage activity and grooming behavior. Mice receiving both PU-H71 and CQ showed fewer symptoms of disease (ruffled fur, lethargy, sunken eyes).

**Table 4:** Summary of the drug combinations tested in the *P.berghei* mouse model trials.

Combinations	Synergy observed (Yes/No)
2.5 mg/kg PU-H71+0.25 mg/kg CQ	No
25 mg/kg PU-H71+0.25 mg/kg CQ	Yes
25 mg/kg PU-H71+0.5 mg/kg CQ	Yes
75 mg/kg PU-H71+0.5 mg/kg CQ	Yes
25 mg/kg PU-H71+5 mg/kg CQ	No
75 mg/kg PU-H71+5 mg/kg CQ	No
25 mg/kg PU-H71+12.5 mg/kg CQ	No
75 mg/kg PU-H71+12.5 mg/kg CQ	No

## Discussion

*Plasmodium falciparum* causes the most severe form of human malaria leading to approximately 0.7–1 million deaths worldwide annually [51]. Eradication is precluded in part by antimalarial drug resistance to several classes of antimalarials. Evolution of resistance to artemisinins [52], [53], the mainstay of treatment for malaria now, bodes poorly for this class of drugs as with previously recommended agents. Combination therapies appear less prone to resistance, but a mechanistic approach for the identification of synergistic agents has not been fully explored in malaria. Many studies show that targeting Hsp90 can be potentially synergistic in various diseases [1], [8]–[10], [12], [18]. The fact that malaria parasites have a life cycle that occurs in two physiologically divergent habitats (cold-blooded mosquito and warm-blooded human host) suggests that a robust adaptation mechanism must exist to manage heat shock caused by the change of host as well as by intense febrile episodes in the human host during severe infection. Therefore, crippling Hsp90, a central player in this response, will inevitably kill the parasite.

Hsp90 has a unique Bergerat fold at the N-terminus that can be inhibited competitively by small molecules [15]. Several studies indicate that Hsp90 clients are essential proteins within abnormal cells [1], [3], [9], [12], [15], [16]. We believe that targeting of cytosolic-inducible PfHsp90 (PF07_0029, new PlasmoDB ID: PF3D7_0827900) is the first and most compelling strategy as this protein appears to be activated under heat shock stress [7] and retains domains consistent with its chaperone function [7], [23], [31]. While others have demonstrated antimalarial properties of inhibiting Hsp90 using a GA derivative [32], our focus was on the synergistic potential of this molecule in recovering the activity of CQ both in vitro and in vivo and understanding the underlying mechanism. Also, because of the toxicity associated with GA and its derivatives, our strategy was to repurpose a novel purine scaffold inhibitor (PU-H71) [19]. PU-H71 is in phase I clinical trials against various neoplastic diseases and has shown no significant toxicity at therapeutic doses [19].

The docking study assumptions were supported by the fact that PU-H71 has high affinity for the PfHsp90 ATP-binding pocket, as demonstrated by the K_d_ constant and inhibition of ATPase activity to levels comparable to that seen with traditional Hsp90 inhibitors such as GA and its derivatives. Tight interaction of PU-H71 with the guanidinium group of R98 may account at least partially for the affinity of PU-H71 for the PfHsp90 ATP binding pocket because the K_d_ constant increased 3.8-fold with the R98K mutation. An alternative explanation for the loss of PU-H71 affinity in the R98K mutant is that this mutation caused a conformational change that disrupted the binding site. In comparison to the IC_50_s observed for PU-H71 with the ATPase assay and in vitro cell cultures, the K_d_ deduced from the SPR studies was high. This may reflect cooperative binding of PU-H71 to the Hsp90 dimer, which may further increase the PfHsp90-PU-H71 interaction affinity. In keeping with our hypothesis and previous studies in *C. albicans* that inhibiting Hsp90 should recoup activity of other key antimalarials, PU-H71 is synergistic with CQ in both susceptible and resistant strains, suggesting that PfHsp90 may be an important, upstream molecular hub in regulating drug resistance. This is akin to the phenotype observed in *Candida albicans* by Cowen and colleagues where reversal of azole resistance was achieved using Hsp90 inhibitors in vitro [3]. In order to determine whether the synergistic phenotype by PfHsp90 inhibitors are related to a physical interaction with putative resistance factors, co-immunoprecipitation studies were carried out. These data demonstrate that a physical interaction occurs between the CQ transporter PfCRT and the PfHsp90 complex. We speculate that disrupting this interaction would lead to early degradation of PfCRT, a decrease in the amount of PfCRT at the digestive vacuole membrane, and consequently a decrease in CQ efflux. An additional key interactor identified for PfHsp90 was the multiple drug resistance transporter 1 (PfMDR1). The gene encoding for PfMDR1 (*pfmdr1)* is amplified in multiple resistant strains of *P. falciparum* including the W2 strain used in this study. The identification of protein degradation machinery proteins as one of the major clusters of the LC-MS/MS analysis from the co-immunoprecipitation hits is consistent with the hypothesis that inhibition of PfHsp90 by PU-H71 would target misfolded PfCRT for degradation (Figure 3E, Table S1). These interaction data are preliminary and further validation studies are required in this apicomplexan parasite. The elevated PU-H71 IC_50_ in the selected Dd2^T163P^ mutant suggests specificity of this purine analog in targeting PfHsp90. We suspect that the essential nature of Hsp90 precludes generation of mutants in the critical ATP-binding domain. The marginal lowering of IC_50_ may be explained by the fact that this mutant Hsp90 molecule is still able to bind ATP for cell survival. A null mutant is almost certainly lethal as observed in other eukaryotic systems [54], [55]. Hence, a knockdown strategy was not attempted. Of concern has been the previous report that although inhibition of the parasite *L.donovani* by geldanamycin was effective, some parasite mutants survived, presumably through episomal amplification of the Hsp90 gene [56]. From a therapeutic standpoint, despite the moderately elevated IC_50_ in response to resistance selection in this study, synergistic activity of PU-H71 with CQ was still observed.

Using an *in vivo* Balb/c mouse model of malaria infection with *P.berghei*, we found a significant effect of PU-H71 in reducing parasitemia and improving survival of mice. Importantly, based on previously published definitions of synergy [39], [44], PU-H71 showed synergistic activity at the doses of 25 mg/kg and 75 mg/kg with sub-therapeutic concentrations of CQ (0.25 mg/kg and 0.5 mg/kg) in vivo. Taken together, PU-H71 is an attractive partner drug for CQ in combination therapy. The lack of any significant toxic effects at doses used for treatment is encouraging.

## Supporting Information

Table S1 **Complete list of constituents of the protein-protein interactome.** The interactions were identified from anti-PfHsp90 and anti-PfCRT immunoprecipitates using LC-MS/MS analysis.
(XLSX)

Table S2 **Summary of the FIC calculations for the synergistic activity of PU-H71 and chloroquine (CQ) in the CQ resistant strain W2.**
(XLSX)
